# “We Should Be Taught Self-Respect, Self-Confidence and Self-Love”: Youth Perspectives of Adult Influences on Their Sexuality and Relationships Among South African Adolescents Living With HIV

**DOI:** 10.3389/frph.2022.913170

**Published:** 2022-07-12

**Authors:** Scarlett Bergam, Caroline Kuo, Millicent Atujuna, Jennifer A. Pellowski, Bulelwa Mtukushe, Nontembeko Ndevu-Qwabe, Mluleki Matiwane, Camerin A. Rencken, Mikaela Belsky, Jacqueline Hoare, Linda-Gail Bekker, Abigail D. Harrison

**Affiliations:** ^1^Brown University School of Public Health, Providence, RI, United States; ^2^Desmond Tutu Health Foundation, Cape Town, South Africa; ^3^Department of Psychiatry and Mental Health, University of Cape Town, Cape Town, South Africa; ^4^Department of Health and Human Biology, Brown University, Providence, RI, United States

**Keywords:** adolescents, South Africa, social support, stigma, structural interventions, linkage to care

## Abstract

**Introduction:**

Of the 1.75 million adolescents aged 10–19 years living with HIV globally, 84% reside in sub-Saharan Africa. This problem is most acute in South Africa, where there are 720,000 adolescents living with HIV (ALHIV). ALHIV navigate the same challenges as other adolescents—such as puberty and first relationships—as well as challenges specific to their HIV-status—including stigma, disclosure, and concerns about HIV transmission. This dual burden calls for tailored sexual and reproductive health (SRH) programs. Here, we qualitatively explore the reflections of South African ALHIV on SRH education, communication, and discussion provided by adults in schools, clinics, and the home related to their unique SRH needs.

**Methods:**

This paper reports on qualitative data from a mixed-methods study to inform interventions that meet the SRH needs of ALHIV. In-depth interviews (*N* = 20) were conducted with ALHIV recruited from two clinics in Cape Town, South Africa. Nine males and 11 females aged 16–19 participated in semi-structured in-depth interviews to discuss their sexual health as ALHIV. The interview guide explored 1) perceived SRH needs; 2) healthy living with HIV; 3) future goals; 4) intimate relationships; 5) psychosocial challenges; and 6) preferred interventions. Data were thematically applied to an iteratively-developed codebook and analyzed by the cross-cultural research team using NVivo 12.

**Results:**

These qualitative data reveal the pressing needs among ALHIV for open communication and accurate information about sexuality and HIV, given the risk to themselves and their partners as they enter intimate relationships. Three themes emerged from the data: 1) Intergenerational pressures coming from caregivers, clinicians, and teachers often stigmatize the sexual heath behaviors of ALHIV; 2) When present, open intergenerational communication can provide ALHIV with crucial information, resources, and social support that supports healthy decisions, and 3) ALHIV offer specific ideas about how adults can support their decision-making in the transition to adulthood.

**Conclusions:**

Findings highlight adolescents' recommendations for programs involving open communication, stigma-reduction around sexuality, and support from both peer and adult mentors. This study lays the foundation for strategies to improve intergenerational communication about sexual health to promote positive approaches to sexuality for ALHIV.

## Introduction

Of the 1.75 million adolescents aged 10–19 years living with HIV globally, 84% reside in sub-Saharan Africa (SSA) (1, 2). Adolescence brings about challenges including puberty, a greater awareness of sexuality, and entry into relationships (3). However, adolescents living with HIV (ALHIV) face added obstacles, including stigma, HIV disclosure, and transmission (4, 5). In order to reach the United Nations' goal of ending HIV as a public health threat by 2030, *The Lancet* calls for more studies interpreting trends in at-risk groups, including adolescents (6). Other calls in the literature note the importance of addressing the health needs of adolescents with empowering and strengthening interventions (4, 7, 8). As a generation of ALHIV transitions into young adulthood, there is a need for qualitative research to deeply explore how ALHIV navigate their sexual decision-making and acquire the needed skills to live successfully with HIV into adulthood.

**Figure 1:**
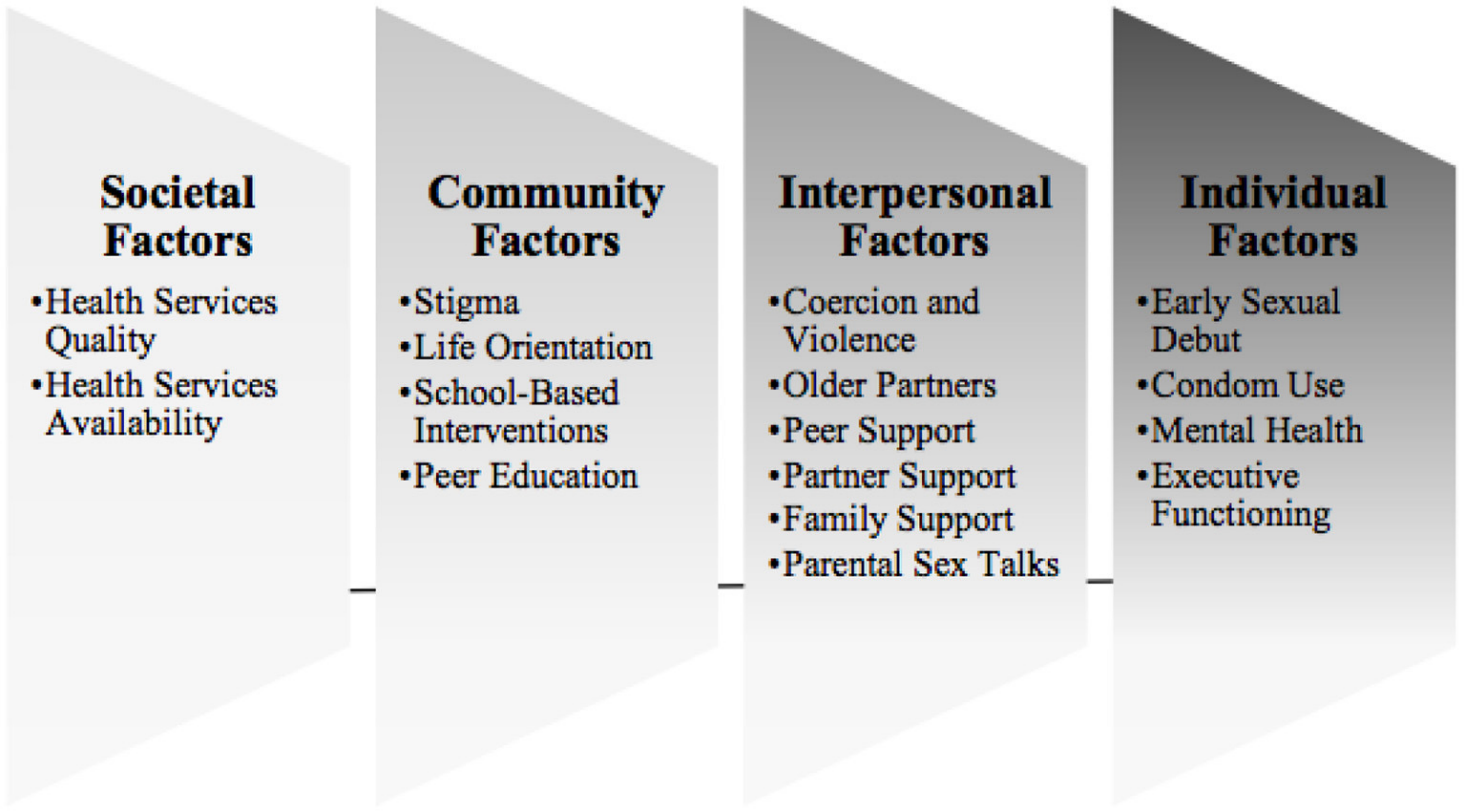
Factors impacting sexual health of ALHIV, overlaid onto the social ecological framework: results of a systematic literature review.

Recent research examines sexual risk behaviors among ALHIV in SSA (8). We use the Socio-Ecological Framework (SEF) as a model to explore the levels of social pressures that influence adolescent health decision-making (9). Figure 1 presents an analysis of findings from quantitative systematic reviews, revealing common risk factors for HIV transmission among ALHIV: early sexual debut, inconsistent condom use, older partners, transactional sex, sex while intoxicated, STIs, sexual violence, and pregnancy (10, 11). ALHIV require effective, multi-level SRH interventions to improve both their sexual health knowledge and behaviors.

Existing research on parental attitudes toward adolescent sexuality in South Africa reveals how cultural norms censor discussions of sexuality between adolescents and parents (12). These norms can contribute to increased sexual activity (13, 14), barriers to preventive behaviors (15, 16), anxiety and misconceptions for ALHIV (17), and discomfort and uncertainty in caregivers (18). Further, the basis for sexuality education in South Africa—Life Orientation (L.O.), a required in-school curriculum for teaching basic life skills—is criticized for being heteronormative and negatively focused, preventing a safe and informed exploration of sexuality (19, 20). L.O has not been shown to lower HIV, STI, or pregnancy rates, but has been found to improve knowledge and the intention to delay sexual activity (21). Randomized trials in SSA (22, 23) and the US (24) have shown that abstinence-only sexual education is ineffective in reducing HIV and STI incidence rates. Although the first meta-analysis on the effectiveness of school-based sexual health interventions in SSA is currently being conducted (25), to date the evidence on effectiveness of such interventions in improving HIV-related outcomes is inconclusive. At the same time, some adults can and do provide meaningful inputs to young people's sexuality education. Adults at home, at school, and at the clinic have unique roles in adolescent sexual education (26, 27). For adolescents living with HIV, parents and other caregivers can provide a crucial support system because they know about an adolescent's HIV status (28). The International HIV/AIDS Alliance encourages meaningful youth engagement rooted in communication, empowerment, respect, trust, and accountability (29). Best practices for empowering young people to address these issues include building self-efficacy, strengthening communication skills, addressing structural barriers, and involving ALHIV in program development (30–32).

One of the most effective approaches to promoting good SRH is specifically tailoring interventions to the complexities of the population of interest (32, 33). For ALHIV, the question remains of how to address the unique needs of this population as they come of age. While many adolescents demonstrate a general working knowledge of HIV and reproductive health, many ALHIV continue to experience large gaps between knowledge and behavior (34–36). Using the SEF to explore multilevel influences on young people's sexual health and related behaviors, this study qualitatively investigates the impacts of intergenerational sexuality education on ALHIV's sexual behaviors in Cape Town, South Africa.

## Methods

### Study Setting

This study took place in the generalized HIV epidemic setting of Cape Town, one of South Africa's three largest cities and the major urban center of the Western Cape Province. Cape Town has a diverse, multi-ethnic population (37), comprising several linguistic groups. The main languages are Afrikaans, isiXhosa, and English (37). Although the Apartheid system ended formally in 1994, there remains the ongoing presence of racialized poverty (38). Many ALHIV are raised by extended family members such as grandparents in residentially segregated neighborhoods. The research site was selected due to the longstanding relationship between our study partner, the Desmond Tutu Health Foundation and one ART clinic in Gugulethu Township, Cape Town, a well-established site providing HIV treatment and care to adolescents and other clients in a supportive setting. ALHIV participants for this study were selected from the clinic site. The qualitative interviews were conducted in a private location within or adjacent to the clinic.

### Study Design and Sample Selection

This study reports data from the qualitative component of a mixed-methods study aimed at developing tailored interventions to address the reproductive health needs of ALHIV. These data are from semi-structured qualitative interviews conducted with male (*n* = 9) and female (*n* = 11) participants aged 16–19 (mean = 18) between July 2018–March 2019.

Twenty ALHIV were recruited for in-depth interviews through convenience sampling by means of informational flyers, followed by an eligibility screening assessment. Criteria for participation included being: 1) 16–19 years and 2) self-reported HIV-positive. All participants provided written informed assent and informed parental consent (if under 18) or written informed consent (if 18 or older). There was no exclusion on the basis of gender, ethnicity, or race, nor on timing of HIV infection. All consent and assent forms were read to the participants in their chosen language, and copies were provided. Informed consent for parents and informed assent for adolescents was obtained after they read the information sheet and had the opportunity to ask questions. Emphasis was placed on the ability to refuse to participate. Each participant received 100 Rand (~$10 at time of interview) and an information resource packet on treatment adherence, mental health, and social services.

### Data Collection

Adolescent interviews lasted approximately 1–1.5 h and took place in a private adolescent program room, conducted by one primary interviewer and an additional research assistant. Demographic data were collected *via* Audio Computer-Assisted Self-Interview Software (A-CASI) using Android smartphones (A-CASI, LLC, Tufts University School of Medicine, 2014). In-depth interviews used a semi-structured agenda with exemplar key questions as “intent statements.” Three co-authors (BM, NN and MM) participated in data collection as well as supervision by one of the South African lead investigators (MA); all were multilingual women. Interviews were conducted in isiXhosa, the primary language of the adolescents. All interviews were audiotaped with a digital voice recorder and audio-transcribed verbatim before being translated into English for analysis. Transcripts were reviewed to remove all personally identifiable information and stored on password-protected computers.

The adolescent interviews followed a semi-structured interview guide that explored six content areas: 1) perceived sexual health needs; 2) healthy living with HIV; 3) future goals; 4) intimate relationships; 5) psychosocial challenges; and 6) preferred interventions.

### Data Analysis

Following translation, transcripts were coded using NVivo 12, a qualitative research software (NVivo/QSR International Pty Ltd. 10.04, Doncaster, Australia). Three US-based researchers developed the initial codebook with review and input from two members of the South African team. The codes were based on the major areas of inquiry for the study such as context (i.e., gender dynamics and socioeconomic status) and intervention needs. The analysis for this paper centered mainly on the two domains of “Intervention Content” and “Context.” Data analysis included the following steps, conducted by two American research assistants: 1) reading and reviewing transcripts, 2) open and axial coding in NVivo, 3) synthesizing codes, and 4) developing preliminary themes. All 20 interview transcripts were double-coded and thematically analyzed with attention to high inter-rater reliability through an iterative, discussion-based process. An audit trail was maintained during data analysis to record decisions for future replicability.

### Ethical Approval

The study was reviewed and approved by the University of Cape Town Health Sciences Research Ethics Committee, South Africa (HREC REF#752/2017) and approved by the Brown University Institutional Review Board (IRB Registration #00000556; Federalwide Assurance (FWA) # 00004460) through the Institutional Authorization Agreement? (IAA #18-15).

## Results

This analysis reveals current adolescent sexual behaviors and their motivations in the context of socio-cultural attitudes and stigma around HIV and sexuality. Adolescents reflect on their needs from adults, including caregivers and clinicians, and suggest changes for adults and for more effective and positive communication about sexual health.

### Intergenerational Pressures Are Profound, and Adult Attitudes Often Stigmatize Sexual Heath Behaviors of ALHIV

Adults are perceived as strict in relation to young people's desire to learn about sexuality and safely entering relationships during the adolescent years. ALHIV participants referenced caregivers, clinical providers, and teachers as trying to exert control over adolescents' relationships. This sentiment was expressed by almost every respondent, generalizing “older people” as “judgmental” about sex.

“You older people are going to ask why we start sex so early. Some of our parents don't understand, like my mother. She's living in the past and not in this democracy of our generation. Even if there's a topic on TV that has something to do with sex, love and pregnancy. I ask her and she won't answer me.” (ID: 8027, Female)

According to adolescents, parents and grandparents frequently expressed negative attitudes toward young people engaging in relationships and sex. One young woman stated that “a parent would shout at you if you talk about sex” (ID: 8026, Female). This idea was repeated by a young female respondent, who also explored the impact of cultural norms about sexuality in her community:

“Black parents are strict, strict for what? My mother is very strict. I have never spoken with her about relationships. But one day I had an STI because I didn't know what to do when having sex, and that's how she knew that I was sexually active.” (ID: 8013, Female)

Young people also faced stigma and judgmental attitudes about sexuality outside the home and family. Many clinicians and counselors, according to one respondent, “just don't feel comfortable talking about sex” (ID: 3003, Female). Another young woman reflected on nurses and other healthcare staff “shouting” and being rude to adolescents in clinics. In combination with concerns about a lack of confidentiality and HIV-related stigma, these stereotypes may deter ALHIV from seeking needed services.

“Most [peers] do not like coming to the clinic, because sometimes nurses can be rude to them. I want to come and prevent they'll be like ‘how can you do that?’, ‘are you sexually active?’ so they feel uncomfortable with it because they ask them as if they can't help them in such a way but they will help them, so it makes them uncomfortable.” (ID: 8009, Female)

In South African schools, L.O. teachers are responsible for providing structured health information to all youth throughout their education. However, respondents frequently mentioned the judgmental and socially stressful atmosphere that prevents students from engaging with the lessons.

“Children are shy and don't want others to see them lifting up their hands to ask questions. I notice that some fall pregnant, but we learnt about it in class so I feel as if it's not enough. We should have another person to talk with who would help without judging.” (ID: 8019, Female)

This respondent expresses a clear need for judgment-free education, or for access to additional resources for advice both in and outside of school. Further, some adolescents were discouraged by the inaccessible words used to describe sexual health in L.O. and how it differs from the way she would like to talk about it.

“Elders will not sugar-coat it, they will put it rudely. They use deep words to describe sex. You become shy to talk about those things.” (ID: 3004, Male)

Ultimately, these adolescent-identified barriers to productive and informative intergenerational conversations about sexual health included scolding, strictness, discomfort, and withholding information, which were encountered at home, in classrooms, and in clinics.

### Open Intergenerational Communication, When Present, Can Provide ALHIV With Accurate Information, Resources, and Social Support

In contrast to the stigma and discomfort reported by many adolescents, some ALHIV reported positive experiences with adults who served as sources of information and support. One adolescent described having some transparency with her mother about her relationship.

“I prefer talking to my mom. I ask for permission when I want to visit my boyfriend because I want her to know my whereabouts. I tell her that I would like to go to my boyfriend, and I will come back soon.” (ID: 8019, Female)

Another respondent acknowledged that introducing partners to parents is a critical part of “serious relationships.” Parental approval is a significant step in establishing strong, long-term relationships with a steady partner whom you “see yourself marrying and having children with.” (ID: 8006, Female)

The adolescent participants in this study were much more likely to seek information about sexuality and relationships from clinical providers than from an older family member. Often, ALHIV have greater access to clinical care than adolescents without HIV, and may have trusted relationships with health care providers, as with this participant:

“All these programs motivate me to be a young adult that is taking her pills and motivating young people to do the same. You motivate me, I take you as my role model because you are just like me and you are working with children like me.” (ID: 8025, Female)

Because these ALHIV are in care and regularly attending clinics to receive ART medications, they experienced greater opportunities for this type of engagement with clinicians. Thus, ALHIV reported that topics covered by clinicians often extend beyond HIV, STI, and pregnancy prevention. One boy explained what he learns during conversations with clinicians: “They say we must use protection, condoms, and not have sex when drunk.” (ID: 8015, Male)

Another teen offered advice from her own personal experiences, highlighting the importance of pregnancy prevention and further noting concerns about parental disapproval.

“[Clinicians] talk with you nicely and ask you questions. They test to see if you're pregnant and they remind you when your appointment is and tell you where to keep your clinic card.” (ID: 8006, Female)

Finally, clinical providers were seen as the best sources of information because their information is perceived as reliable and correct, as one teen said: “It's more comfortable with a person whose profession is dealing with those things.” (ID: 8009, Female)

Despite concerns expressed about L.O. by the adolescent participants, adolescents also acknowledged that L.O. teachers were more comfortable talking about sexuality than parents. They perceived teachers as more comfortable talking about sex and informing students about methods for prevention beyond abstinence alone. One respondent discussed how her peers learned about sexuality in school “because their parents haven't done the sex talk.” (ID: 8006, Female). Another adolescent described how her peers are comfortable asking for information from teachers:

“[My peers] feel comfortable with L.O. teachers. They feel free responding to questions. They would ask if we have boyfriends and if we are having sex with them. They teach us about pregnancy and advise us about contraception methods, HIV, and STIs.” (ID: 8019, Female)

These adolescents reflected on the importance of accurate and detailed prevention information in addition to the empowering and positive attitudes of teachers and other adults.

### Adolescents Want and Need the Adults in Their Lives to Provide Support to Empower Them to Make Healthy Decisions Despite Negative External Pressures

While most adolescents offered both positive and negative perspectives on the resources available to them, few were able to articulate a different way of providing information about health, sexuality, and prevention. Many addressed the known gaps by stating simply that they needed more: “I think I still need more information” (ID: 3009, Female). Another adolescent puts very simply the need for “support and care” from “doctors, nurses and counselors” at the clinic (ID: 8022, Female). Despite the stated problems, some adolescents maintained the idea that “improving the Life Orientation curriculum” would be best (ID: 8016, Male).

As young people living with HIV, there was also a recognition of the need for psychosocial and mental health support as a component of any program. One young woman described her vision for how all adults in the lives of ALHIV could contribute to their empowerment through psychosocial support to build resilience, arguing that such programs should go hand in hand with traditional informational components of sexuality education.

“These children know these things. They should be taught self-respect, self-confidence and self-love. They shouldn't rush things in life.” (ID: 8017, Female)

Suggestions like these provide a step toward ALHIV becoming mentally strong young adults capable of making healthy decisions. Representing these ideals, a female participant models positive HIV status disclosure between a parent and child.

“If I had a child and my child was 10 years old, I would say: […] ‘Listen child, don't get shocked as to what I'm going to say. Just accept it and continue with it. Firstly, what I'm going to say won't kill you if you are doing the right things, secondly, if you are doing the right things you would live forever, there isn't a certain age that you will die, [you] can become 100 and live with HIV. You can cry if you want to but your tears won't help you because […] you must treat it well in order for your life to be normal.” (ID: 8012, Female)

Ideally, intergenerational and psychosocially supportive discussions should take place early and frankly. When disclosing to children their HIV status, caregivers and clinicians can enforce the idea that living with HIV is not a death sentence, nor is it an entire identity. Here, one adolescent discusses the need for adults to prioritize her mental wellness and individual autonomy while providing sexual health education and resources.

“I don't think I can be passionate about what I want. I am not strong enough emotionally. Physically, I know I am strong, but I just need support emotionally.” (ID: 8027, Female)

These adolescents' lived experiences can and should serve as a guide to facilitate intergenerational, comprehensive sexual health education that meets the unique needs of ALHIV. Youth living with HIV seek out sexual health empowerment through grounding, adolescent-friendly interventions and positive communication with adult role models.

## Discussion

In this qualitative study, adolescents shared their perspectives on how intergenerational pressures impact their ability to make safe decisions regarding sexual activity, widening the gap between adolescents' knowledge and their use of safer sex practices. Adolescents presented their memorable experiences with both shaming and empowering adult role models, which can serve as a guide for positive intergenerational communication about sexuality. Parents, clinicians, and L.O. teachers have the potential to be a primary source of comprehensive sexuality education for young people. Most adolescents, regardless of HIV status, face negative attitudes from adults that reduce their access to sexual health resources. This discouragement is especially risky for ALHIV, who face additional risks for themselves and their partners in sexual relationships, and has led the ALHIV in our study and their peers to face STIs and pregnancy at an early age. While many ALHIV are unaware of their status, most diagnosed ALHIV are already linked to regular care and have access to information and some supportive clinicians (7, 32, 39). Additional support, particularly psychosocial and mental health support, can help ALHIV to address difficulties they face individually and within their families and communities, and to overcome past traumas, and should include resources and education to cover all aspects of sexual health and healthy relationships. In these findings, young people's understanding of their need for psychosocial support and mental health interventions is a profound reminder of the gaps they face.

Some ALHIV exhibited a sex-positive, healthy mindset around romantic and sexual relationships by discussing intrinsic empowerment, family support, positive self-image, long-term goals, and social independence. With the help of supportive adults who have helped to shape their self-image, some ALHIV felt confident in their ability to make healthy choices around relationships and sexual activity—whether through delaying sexual activity until ready for relationships, or through safer sex and relationship practices. The school and the clinic are crucial mechanisms for the encouragement of sex-positive, well informed, and healthy lifestyles, but often fall short (21, 40–42). Systematic training for ALHIV-targeted sexuality education is required to tailor programs to their specific needs. The lived experiences of ALHIV can serve as the basis for interventions that give adults the tools and vocabulary to discuss sensitive topics.

Existing quantitative (23, 43–46) and qualitative (47–49) studies on adolescent SRH needs in SSA, such as this one, demonstrate the gaps in young people's access to information and resources related to SRH, and provide the basis for the argument that sexuality education must be provided to younger adolescents. Importantly, these findings confirm research from the past decade, highlighting the magnitude of work that remains to be done to more effectively address the SRH needs of ALHIV (42, 50–52). Recent interventions highlight the importance of family engagement (29, 51, 53), peer-based approaches including peer support groups (8, 52), and newer ways of engaging youth, such as mHealth (30, 43, 49, 54). One model program that does exist to improve intergenerational communication with ALHIV about their SRH needs in SSA is the Families Matter! Program (52). Currently, many adolescents receive partial information through school-based or other established programs, signaling the critical need to enhance these standard approaches with more comprehensive and youth-friendly programs. Even when information is accessible and available, multi-level social pressures highlighted offer challenges to the adoption of healthy sexual behaviors. Reflecting on the contributions of the study framework, the socioecological model highlights how multilevel factors influence these findings. Adolescent SRH behaviors like condom use are influenced by adolescent mental health (individual factor), parental sex talks (interpersonal factor), quality of Life Orientation (community factor), and societal attitudes (societal factor). Interventions should offer support at each of these levels, helping ALHIV to make the decisions they know are healthy, especially when they require difficult conversations, constant self-reflection, and resisting peer pressures. The qualitative findings from this study map onto these patterns, where adolescent reflections on their own decision-making balance self-perception and internal motivation with perceived support and access to outside resources.

This theoretical framework provides practical insight into interventions likely to be effective with this group, simultaneously engaging youth and impactful adults in their support networks (10), and illustrating specific recommendations for mapping onto existing intervention approaches. Specifically, these interventions could take the form of mHealth or in-person interventions with parallel programming to simultaneously engage ALHIV, caregivers, and clinicians, targeting all levels of the socio-ecological framework (41, 50, 51, 53–56). There is also a need for future interventions to focus on positive parenting around sexuality, integrated HIV and mental healthcare, communication and decision-making skill-building, and socially acceptable health education to encourage healthy sexual decision-making in ALHIV. Importantly, adolescents want to, and should, be actively engaged in all stages of research and intervention development pertaining to their unique SRH needs, in line with best practice recommendations in the literature.

## Limitations

Many of the adolescents in this study are linked to HIV care, which neglects the populations that do not know their status or that have limited treatment availability. With many perinatally infected adolescents in the study, most have known of their HIV status for some time. Our study did not differentiate results between perinatally and behaviorally-infected adolescents. Adolescents in more resource-limited settings face further struggles with disclosure, fewer resources, and risky sexual behaviors due to lack of awareness of their status and more limited access to care. Concurrent analyses from this study examine other perspectives, including adult role models and clinicians, and topics beyond intergenerational communication.

## Conclusions

This study's findings ask: what must be changed about current SRH education to support healthier decision making for ALHIV, and how can other interventions address these needs? ALHIV yearn for empowering, transparent relationships with adults long before becoming sexually active, encouraging adults to guide healthy decision-making given their unique life and social circumstances. Additional support beyond their current linkage to clinical care, including more resources for psychosocial and mental health support, can help ALHIV to address difficulties they face individually and within their families and communities. Ultimately, supporting ALHIV in South Africa will require encouragement, empowerment, and open communication among ALHIV through interventions that center young voices.

## Data Availability Statement

The datasets generated during the current study are available by contacting corresponding author, at the discretion of the study team.

## Ethics Statement

This study involving human subjects was reviewed and approved by the Institutional Review Board (IRB) at Brown University and the Health Sciences Research Ethics Committee of the University of Cape Town, South Africa. Written informed consent to participate in this study was provided by the participants for those 18 and above, or the participants' legal guardian/next of kin.

## Author Contributions

SB has first authorship of this article. AH has sole senior authorship as an editor and advisor throughout the writing process. JH, L-GB, and CK served as Co-PIs on the parent study. JP, CR, and MB share equal co-authorship as secondary editors of this project. MA, BM, NN-Q, and MM share equal co-authorship as those who led data collection and project implementation. All authors contributed to the article and approve the submitted version.

## Funding

This study is funded by the U.S. National Institutes of Health (Eunice Kennedy Shriver National Institute of Child Health and Human Development R21HD089825). Through Brown University, the primary author also received the 2019-20 Barbara Anton Community Research Grant for the composition of this manuscript.

## Conflict of Interest

The authors declare that the research was conducted in the absence of any commercial or financial relationships that could be construed as a potential conflictof interest.

## Publisher's Note

All claims expressed in this article are solely those of the authors and do not necessarily represent those of their affiliated organizations, or those of the publisher, the editors and the reviewers. Any product that may be evaluated in this article, or claim that may be made by its manufacturer, is not guaranteed or endorsed by the publisher.

## Abbreviations

ALHIV: Adolescents Living with HIV
ART: Anti-Retroviral Treatment
A-CASI: Audio Computer-Assisted Self-Interview Software
CSE: Comprehensive Sexuality Education
HIV: Human Immunodeficiency Virus
IRB: Institutional Review Board
L.O.: Life Orientation
SEF: Socio-Ecological Framework
SSA: Sub-Saharan Africa.
